# Multiple Sclerosis: Switching from Natalizumab to Other High-Efficacy Treatments to Mitigate Progressive Multifocal Leukoencephalopathy Risk

**DOI:** 10.1007/s13311-021-01102-w

**Published:** 2021-09-03

**Authors:** Hans-Peter Hartung, Jan Mares, Sven G. Meuth, Thomas Berger

**Affiliations:** 1 Department of Neurology, Heinrich-Heine-University Düsseldorf, Düsseldorf, Germany; 2 Brain and Mind Centre, University of Sydney, Sydney, Australia; 3 Department of Neurology, Palacky University, Olomouc, Olomouc, Czech Republic; 4 Department of Neurology, Medical University of Vienna, Vienna, Austria

With the approval of the injectables interferon-β and glatiramer acetate a quarter of a century ago, the new era of multiple sclerosis (MS) treatment started [1]. A decade passed until the first representative of the second generation of immunomodulatory drugs for MS emerged: natalizumab. The evolution from unravelling the mechanisms of lymphocycte homing and migration during inflammatory processes, identification of critical molecular checkpoints, experimental blockade of decisive molecular interactions in vitro and in the animal model of experimental autoimmune encephalomyelitis by a monoclonal antibody, and translation into therapeutic trials in people with MS can serve as a case study of successful rational drug development [2, 3]. The recombinant humanized monoclonal antibody natalizumab recognizes the alpha4 subunit of integrins that is expressed predominantly on T lymphocytes but also by B lymphocytes and neutrophils. Natalizumab disrupts the interaction between alpha4 integrin and very late antigen VLA-4 displayed on the surface of endothelial cells. Consequently, invasion of the CNS through the blood–brain barrier by potentially autoaggressive T cells is diminished or blocked [2, 4]. Phase 2 and 3 clinical trials and extensive real-world experience underscored the high efficacy of natalizumab, which compared to the first-generation treatments had the additional advantage of requiring less frequent administration and exhibiting overall good tolerability and safety [3]. The most dreaded complication is progressive multifocal leukoencephalopathy (PML) [5]. Approximately 25% of patients developing PML following natalizumab therapy die and a large proportion are left with marked disability. A risk stratification scheme has been implemented accounting for JC virus antibody index, treatment duration, and prior exposure to immunosuppressant drugs [5, 6]. Strict adherence has apparently reduced the number of natalizumab users who have come down with PML [7, 8]. The main reason driving the decision to discontinue the highly effective immunomodulator natalizumab is the potential risk to develop PML as signified by a high JC virus antibody index and reaching a critical threshold of 18 months of continued natalizumab administration [9, 10]. Stopping natalizumab treatment has been associated with disease reactivation, which cannot be sufficiently prevented by subsequent use of lower efficacy drugs [11, 12]. Avoiding disease recrudescence clearly is of fundamental clinical importance. It requires appropriate timing of the interval between stop and commencement of a new disease modifying treatment, taking into account pharmacokinetics and pharmacodynamics of natalizumab, in particular saturation of alpha4 integrin on lymphocytes, and the choice of a high-efficacy drug that can contain pathobiological and clinical MS activity with an acceptable safety profile. Based on the results of pivotal trials and real-world experience, the anti-CD20 monoclonal antibodies ocrelizumab and rituximab [13–19] and oral cladribine [20–22], a synthetic purine analogue, are accepted as highly efficacious and overall safe treatments for relapsing MS [13–22].

As published in the April 2021 issue of *Neurotherapeutics*, a retrospective observational study representing a joint effort of 11 Italian MS centers compared effectiveness, tolerability, and safety of switching disease-modifying treatment in relapsing MS patients with a high JCV antibody index and at least 24 infusions (administered monthly for at least 1 year and then with standard or extended interval) from natalizumab to ocrelizumab, rituximab, or cladribine [23]. The primary outcome was the annualized relapse rate with additional outcome MRI activity after 12 months and 12 weeks confirmed disability progression. The investigators applied a generalized regression model using treatment as independent variable and age, sex, EDSS in the year before treatment switch, number of natalizumab infusions, and EDSS during natalizumab treatments as covariates. Of 120 patients fulfilling inclusion criteria, 64 switched to ocrelizumab, 36 to rituximab, and 20 to cladribine. The mean annualized relapse rates in these groups were 0.001, 0.308, and 0.5000, respectively. Patients who switched to ocrelizumab had a lower risk for MRI activity. There was no difference in confirmed disability progression. No PML occurred. Severe infections were reported in 3 patients on ocrelizumab, one on rituximab, and one on cladribine. A recent smaller study from one Italian center similarly looked at efficacy and safety of switching natalizumab users deemed to carry an elevated risk of developing PML (duration of exposure and JCV antibody index) to ocrelizumab [24]. In the first 3 months, in one of 42 patients a relapse and in 4 individuals MRI activity were recorded, whereas in the subsequent 3 months, no relapse occurred. Similar control of disease activity was reported in 2 retrospective studies from the USA and Germany involving 28 and 20 patients, respectively [25, 26].

A recent observational study from Amsterdam analyzing 42 patients who stopped natalizumab and switched directly or indirectly to ocrelizumab obtained no evidence of disease activity (NEDA)-3 in 83% or 50%, respectively [27]. Two patients who received ocrelizumab directly had carry-over mild PML [28], an infrequent event in general, observed also with fingolimod.

In a multicenter Swedish study of 256 relapsing MS patients who discontinued natalizumab because of JCV antibody positivity, rituximab was markedly superior to fingolimod in keeping clinical and MRI activity at bay over a period of 18 months [29]. One single small study of 17 patients stopping natalizumab because of high JCV antibody index (*n* = 13), continued disease activity (*n* = 6), presence of MRI disease activity (*n* = 4), and a switch to oral cladribine demonstrated effective disease suppression over a mean period of 9.7 months and no serious adverse events other than the expected lymphopenia [30].

In aggregate, these observational studies provide evidence that high-efficacy drugs are effective and generally safe in a critical situation of MS management when treatment with natalizumab is discontinued. The results of an ongoing multicenter prospective open-label phase IV study examining the transition from natalizumab to ocrelizumab (interval 4–6 weeks) are expected by mid-2022 (Clinicaltrial.gov identifier NCT03157830) and will yield further data on which to base the important therapeutic decision.

Finally, pilot studies reported abolition or marked diminution of PML risk in individuals receiving natalizumab at extended dosing intervals [31, 32]. These findings prompted a number of ongoing observational and controlled randomized trials investigating feasibility, effectiveness, and safety of such an approach, with the particular goal to further minimize the risk of PML (Clinicaltrial.gov identifier: NCT04225312; NCT04580381; NCT0368992; NCT03516526). Results are eagerly awaited and will undoubtedly be implemented in current treatment algorithms.

## Supplementary Information

Below is the link to the electronic supplementary material.

Supplementary file1 (PDF 508 KB)

Supplementary file2 (PDF 508 KB)

Supplementary file3 (PDF 508 KB)

Supplementary file4 (PDF 508 KB)

## Declarations

SGM received honoraria for lecturing and travel expenses for attending meetings from Almirall, Amicus Therapeutics Germany, Bayer Health Care, Biogen, Celgene, Diamed, Genzyme, MedDay Pharmaceuticals, Merck Serono, Novartis, Novo Nordisk, ONO Pharma, Roche, Sanofi-Aventis, Chugai Pharma, QuintilesIMS, and Teva. His research is funded by the German Ministry for Education and Research (BMBF), Deutsche Forschungsgemeinschaft (DFG), Else Kröner Fresenius Foundation, German Academic Exchange Service, Hertie Foundation, Interdisciplinary Center for Clinical Studies (IZKF) Muenster, German Foundation Neurology, and by Almirall, Amicus Therapeutics Germany, Biogen, Diamed, Fresenius Medical Care, Genzyme, Merck Serono, Novartis, ONO Pharma, Roche, and Teva.
